# The complete plastid genome sequence of *Viola selkirkii* Pursh ex Goldie (Violaceae)

**DOI:** 10.1080/23802359.2022.2090299

**Published:** 2022-06-28

**Authors:** Ah-reum Go, Kyeong-Sik Cheon, Ki-Oug Yoo

**Affiliations:** a Department of Biological Sciences, Kangwon National University, Chuncheon, South Korea; b Department of Biological Science, Sangji University, Wonju, South Korea

**Keywords:** Plastome, plastid genome, Violaceae, *Viola selkirkii*, phylogenetic analysis

## Abstract

*Viola selkirkii*, belonging to the genus *Viola*, has heart-shaped leaves and pale purple flowers, and it is widely distributed in the Northern Hemisphere, including Europe, North America, and Asia. In this study, the plastid genome of *V. selkirkii* was sequenced and phylogenetic analysis was performed on 11 *Viola* plastid genomes. The length of the plastid genome length of *V. selkirkii* was 156,774 bp, and it was identified as having a typical quadripartite structure with a large single-copy region (85,930 bp), a small single-copy region (17,982 bp), and two inverted repeat regions (26,431 bp each). A phylogenetic analysis was conducted with 77 protein-coding genes from the complete plastid genomes of 11 *Viola* and nine Salicaceae species; the complete plastid genome of *Erythroxylum novogranatense* was used as an outgroup. *Viola* formed a monophyletic clade, and *V. selkirkii* was closely related to *V. ulleungdoensis*. These results contribute to the clear identification of the phylogenetic position of *V. selkirkii* in *Viola.*

Violaceae Batch., which contains *Viola selkirkii* Pursh ex Goldie 1822, includes herbaceous, shrubs, and small shrubs and consists of 22 genera, and approximately 1000–1100 species (Wahlert et al. 2014). The largest genus, *Viola* L., contains approximately 580–620 species, and most of them are distributed in the Northern Hemisphere (Clausen 1964; Yoo and Jang 2010; Wahlert et al. 2014; Cheon et al. 2020). *V. selkirkii* is widely distributed in the Northern Hemisphere, including Europe, North America, and Asia, and its habitat is shaded, cold mountain forests and moist soils at high latitudes (Brainerd 1921; Russell 1956; Kim 1986). *V. selkirkii* is recognized as a member of *Viola* sect. *Plagiostigma* ser. *Estolonosae* (Marcussen et al. 2012). A morphological study of *V. selkirkii* was conducted by Russell (1956). He observed specimens in North America and found that the leaves were slightly wider toward the west, but the difference was not sufficient to distinguish a species (Russell 1956). In this study, the plastid genome of *V. selkirkii* was determined for the first time, and phylogenetic analysis was performed to identify the relationships within *Viola.*

DNA was extracted using fresh leaves from Mt. Il-san, Gangwon-do Province (N38° 11′ 17.0″, E127° 47′ 48.0″). The voucher specimen was deposited at the Kangwon National University Herbarium (KWNU, https://biology.kangwon.ac.kr/biology, Ki-Oug Yoo, yooko@kangwon.ac.kr) under the voucher no. KWNU 98801. DNA extraction was performed using a DNA Plant Mini Kit (Qiagen Inc., Valencia, CA). The extracted DNA was sequenced using the Illumina MiSeq platform (Illumina Inc., San Diego, CA), and paired-end reads with an average read length of 301 bp were identified from the 3,364,589 raw reads. We used the Map to Reference function in Geneious 7.1.9 (Biomatters Ltd., Auckland, New Zealand) to exclude nuclear and mitochondrial reads based on the published plastid genome of *V. seoulensis* Nakai 1918 (GenBank accession no. KP749924). Then, *de novo* assembly was implemented using Geneious 7.1.9, and a total of 378,781 reads were aligned. The complete plastid sequence was annotated based on the online program GeSeq (Tillich et al. 2017) coupled with manual correction for the start and stop codons. Additionally, we referred to published *Viola* plastid genomes (GenBank accession nos. KP749924, MH229816, MH229819, and MT012304).

The complete plastid genome of *V. selkirkii* (GenBank accession no. MW448361) has a total length of 156,774 bp (GC content: 36.3%). This genome has a typical quadripartite structure with a large single-copy (LSC) region of 85,930 bp, a small single-copy (SSC) region of 17,982 bp, and two inverted repeats (IRs) of 26,431 bp each. The plastid genome of *V. selkirkii* has a total of 131 genes, with 84 protein-coding genes, 37 tRNA genes, eight rRNA genes, and two pseudogenes. The IR regions contain 17 duplicated genes (*ndhB*, *rpl2*, *rpl23*, *rps7*, *rps12*, *ycf2*, *ycf15*, *rrn16*, *rrn23*, *rrn4.5*, *rrn5*, *trnA-UGC*, *trnI-GAU*, *trnL-CAA*, *trnN-GUU*, *trnR-ACG*, and *trnV-GAC*). Within the plastid genome of *V. selkirkii*, eight protein-coding genes (*atpF*, *ndhA*, *ndhB*, *petB*, *petD*, *rpl2*, *rpl16*, and *rpoC1*) and six tRNA genes (*trnA-UGC*, *trnG-UCC*, *trnI-GAU*, *trnK-UUU*, *trnL-UAA*, and *trnV-UAC*) have one intron each, and three genes (*clpP*, *rps12*, and *ycf3*) include two introns.

To determine the phylogenetic location of *V. selkirkii* in *Viola*, we conducted a phylogenetic analysis. A total of 77 protein-coding genes from 11 Violaceae species, nine Salicaceae species, and one outgroup (*Erythroxylum novogranatense*) were aligned by MAFFT (Katoh et al. 2002). Phylogenetic analyses were conducted with the maximum-likelihood (ML) method using RAxML v.7.4.2 with 1000 bootstrap replicates and the GTR + I model (Stamatakis 2006). The intraspecific classification system followed a study by Marcussen et al. (2012). The phylogenetic analysis results (Figure 1) showed that *Viola* had a strongly supported monophyly (BS = 100) and formed a clade distinct from Salicaceae. *V. selkirkii* was closely related to *V. ulleungdoensis* and was placed in a basal position within sect. *Plagiostigma*. Our results contribute to the clear identification of the phylogenetic position of *Viola* and the phylogenetic relationships of other genera within Violaceae.

**Figure 1.**
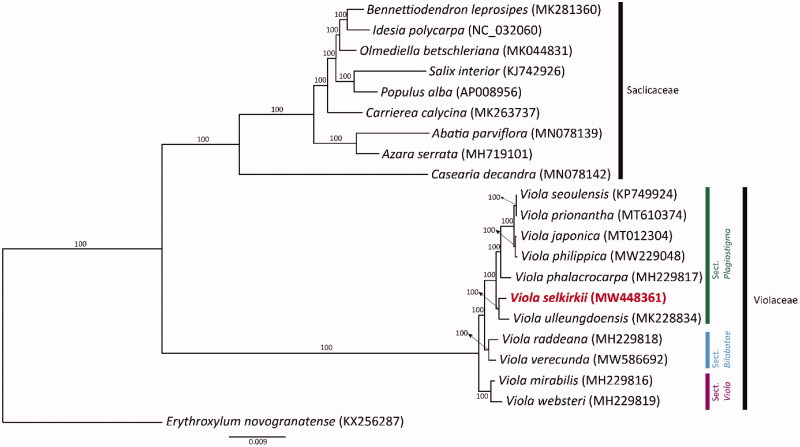
ML tree based on 77 protein-coding genes from the complete plastid genomes of 11 *Viola* species, nine Salicaceae species, and one outgroup. Each GenBank accession number is indicated next to the species name. The bootstrap values are displayed above the branch nodes.

## Data Availability

The genome sequence data that obtained at this study are openly available in GenBank of NCBI (https://www.ncbi.nlm.nih.gov/) under the accession number MW448361. The associated BioProject, SRA, and Bio-Sample numbers are PRJNA817383, SRR18352840, and SAMN26764828, respectively.
